# The Prevalence, Etiology and Treatment of Gastroduodenal Ulcers and Perforation: A Systematic Review

**DOI:** 10.3390/jcm13041063

**Published:** 2024-02-13

**Authors:** Rizki Amalia, Amie Vidyani, Reny I’tishom, Wiwin Is Efendi, Edwin Danardono, Bogi Pratomo Wibowo, Muhammad Lutfi Parewangi, Muhammad Miftahussurur, Hoda M. Malaty

**Affiliations:** 1 Department of Environmental and Preventive Medicine, Oita University Faculty of Medicine, Yufu 879-5593, Japan; rizkiamalia.kia93@gmail.com; 2 *Helicobacter pylori* and Microbiota Study Group, Institute of Tropical Disease, Universitas Airlangga, Surabaya 60286, Indonesia; muhammad-m@fk.unair.ac.id; 3 Division of Gastroentero-Hepatology, Department of Internal Medicine, Faculty of Medicine-Dr. Soetomo Teaching Hospital, Universitas Airlangga, Surabaya 60115, Indonesia; 4 Department of Medical Biology, Faculty of Medicine, Universitas Airlangga, Surabaya 60132, Indonesia; ritishom@fk.unair.ac.id; 5 Department of Pulmonology and Respiratory Medicine, Faculty of Medicine, Universitas Airlangga, Surabaya 60132, Indonesia; wisepulmo@gmail.com; 6 Department of Surgery, Faculty of Medicine, Universitas Airlangga, Surabaya 60132, Indonesia; edwin.digestive@gmail.com; 7 Department of Endocrinology, Saiful Anwar General Hospital, Malang 65111, Indonesia; bogi.pratomo@yahoo.com; 8 Division of Gastroenterology-Hepatology, Department of Internal Medicine, Faculty of Medicine, Hasanuddin University, Makassar 90245, Indonesia; iparewangi@gmail.com; 9 Department of Medicine, Baylor College of Medicine, Houston, TX 77030, USA

**Keywords:** gastroduodenal, perforation, *Helicobacter pylori* infection, NSAIDs, humans and disease

## Abstract

(1) Background: Gastroduodenal perforation (GDP) is a life-threatening condition caused by a spontaneous or traumatic event. Treatment should be based on the mechanism of damage, timing, location, extent of the injury, and the patient’s clinical condition. We aimed to examine several etiologic factors associated with gastroduodenal perforation and to search for the best method(s) for its prevention and treatment. (2) Methods: We conducted extensive literature reviews by searching numerous studies obtained from PubMed, Science Direct, and Cochrane for the following keywords: gastroduodenal perforation, *Helicobacter pylori*, NSAIDs’ use, side effects of GDP, laparoscopy, and surgery. The primary outcome was the reported occurrence of GDP. (3) Results: Using keywords, 883 articles were identified. After applying the inclusion and exclusion criteria, 53 studies were eligible for the current analyses, with a total number of 34,692 gastroduodenal perforation cases. Even though the risk factors of gastroduodenal perforation are various, the prevalence of *H. pylori* among patients with perforation is considerably high. As technology develops, the treatment for gastric perforation will also improve, with laparoscopic surgery having a lower mortality and complication rate compared to open surgery for GDP treatment. (4) Conclusions: *H. pylori* infection plays the most significant role in GDP, more than NSAIDs, surgery, chemotherapy, or transplantation. Treatment of *H. pylori* infection is essential to decrease the prevalence of GDP and speed up its recovery. However, urgent cases require immediate intervention, such as laparoscopic or open surgery.

## Highlights

While (NSAIDs) have a greater risk of inducing GDP, the occurrence of NSAID-induced GDP is relatively low compared to the incidence of *H. pylori* infection.

**Main Findings**

- The presence of *H. pylori* infection is strongly associated with the development of GDP.
- It is imperative to eliminate the infection to halt its progression and expedite the recovery of GDP.

**Implications of the main Findings**

- Treatment of *H. pylori* is vital for GPD patients.
- Patients with sever complications due to GPD must have laparoscopic or surgery.

## 1. Introduction

Duodenal and gastric ulcers remain the two most common perforations of the gastrointestinal tract. A gastric ulcer refers to a specific area of inflammation or damage on the lining of the stomach, whereas gastric perforation is a more serious issue characterized by the development of a hole or tear in the wall of the stomach. Gastric perforation commonly occurs as a consequence of untreated or inadequately controlled gastric ulcers. Both disorders necessitate medical intervention, but stomach perforation is regarded as a medical emergency owing to its capacity for severe consequences [1]. Risk factors of gastroduodenal perforation (GDP) vary based on the geographical area associated with sociodemographic and environmental factors, such as overcrowding and poor hygiene in developing countries, and it mostly occurs spontaneously due to peptic ulcer disease [2]. The mean prevalence of *H. pylori* infection in patients with perforated peptic ulcers is roughly around 65–70%, and this might indicate that there are more factors involved in its pathology [3]. In many countries, the incidence of *H. pylori* infection has been decreasing in association with an improved standard of living [4]. Patients often fails to perceive the symptoms and indicators of GDP, resulting in delayed treatment and jeopardizing their lives. Therefore, the use of CT scans is very important and needs to be considered as an effort to improve the ability to detect perforation [5].

The incidence and prevalence of GDP in recent years have attracted the attention of researchers in the medical field. Interest has always focused on *H. pylori* eradication as a strategy for eliminating gastroduodenal perforation. The objective of this study is to determine the most efficacious approach(es) for preventing and treating gastroduodenal perforation while also examining several etiological factors associated with this illness. Our research carries out further efforts into realizing the Sustainable Development Goals (SDGs) in Indonesia and ensuring a healthy life and good welfare for all people of all ages. 

## 2. Methods

### 2.1. Literature Search and Study Selection

This systematic review was performed using the PRISMA (Preferred Reporting Items for Systematic Reviews and Meta-Analyses) 2020 guidelines [6]. A search was completed in January 2022 using keywords from a combination of Medical Subject Headings in PubMed, Cochrane, and Science Direct with several inclusion and exclusion criteria (Table 1). The search terms used were “Gastroduodenal” and “Perforation” filtered with clinical trials and randomized controlled trial studies. Additionally, the reference lists of the selected articles were manually reviewed to obtain other potentially relevant articles. Selected publications were all in the English language. 

### 2.2. Data Extraction and Quality Appraisal

**Figure 1:**
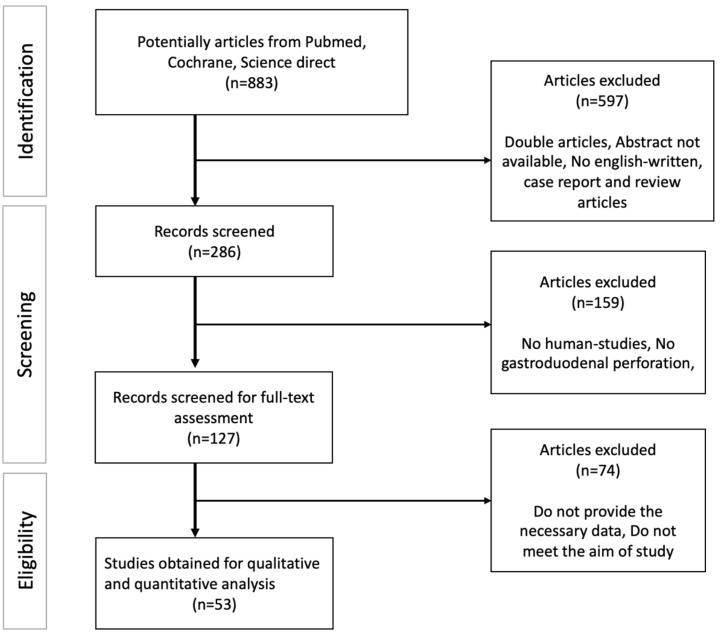
Flowchart of PRISMA diagram.

The flowchart of the articles obtained in this study is shown in Figure 1. There were 883 articles identified after applying the keywords, of which 597 articles were excluded after screening because they did not meet the criteria. Abstract and full-text assessments were conducted to obtain relevant data; the final 53 eligible articles were used in this study. 

### 2.3. Data Analysis

We divided the eligible articles for the current analysis into three categories based on the cause of perforation: *H. pylori* infection, NSAID treatment, and other risk factors for GDP. We extracted the total number of patients and calculated the prevalence of the perforation among each of the three groups independently.

## 3. Results

### 3.1. Gastroduodenal Perforation Event and Prevalence

A total of 53 studies were qualified to acquire data, with a total of 118,600 patients obtained in this study. Out of a total of 118,600 patients included in this study, 34,692 individuals experienced gastroduodenal perforation, and 4666 patients passed away. Moreover, 12 of the 53 articles obtained tested participating patients for *H. pylori*, while the other studies did not disclose the infection.

### 3.2. The Prevalence of H. pylori Infection among Patients with Gastroduodenal Perforation

The overall prevalence of *H. pylori* infection among the total 1653 cases of perforation was 58% (Table 2). That prevalence varied between the studies from 3% to 100%, and this difference could be due to the wide variation of the sample size and/or the differences in the diagnostic methods used. There were 5 studies with a sample size greater than 100 patients vs. 7 studies with less than 100 patients. The overall prevalence of *H. pylori* was significantly higher among the group that participated in the larger sample size studies (61% vs. 39%, respectively, *p* = 0.0001). Studies by Pescatore et al. [7] and Tokunaga et al. [8] showed the highest *H. pylori* prevalence among patients with GDP and ranged from 92% to 100% (*n* = 119 patients), compared to the other studies that showed a prevalence of infection between 20 and 84.6% (*n* = 1457) [9,10,11,12,13,14,15,16]. Two of the studies reported the lowest prevalence of *H. pylori* infection, with 12.5% among a total number of 48 patients and 13.8% among 29 patients with a perforated peptic ulcer, which could be due to the small sample size of both studies [3,17].

### 3.3. NSAIDs’ Use among Gastroduodenal Perforation

Non-steroidal anti-inflammatory medicines (NSAIDs) are a pharmacological category frequently used for pain relief, inflammation reduction, and fever reduction. They function by suppressing the activity of an enzyme known as cyclooxygenase (COX), which has a crucial role in the synthesis of prostaglandins, which have a role in the promotion of inflammation, pain, and fever inside the body [18]. Table 3 shows the prevalence of GDP among NSAID users according to the type of NSAID used. Among the total number of 8076 patients who used rofecoxib and naproxen, the risk ratio for perforation was 0.40 [19]. The reported risk ratios among patients using celecoxib, ibuprofen, naproxen, and other NSAIDs slightly differ: 0.44, 0.48, and 0.49, respectively [12,20,21]. The risk ratio below 1 indicates that the risk of GDP among the exposed group was relatively low [19]. However, studies of NSAIDs in Japan demonstrated a very high risk ratio of 1.715 [21]. In patients with rheumatoid arthritis, rofecoxib, a selective cyclooxygenase-2 inhibitor, shows fewer upper gastrointestinal events than naproxen. Celecoxib, compared to ibuprofen and naproxen, reduces clinically significant gastrointestinal events, but its benefits decrease with low-dose aspirin. Long-term NSAID use increases the prevalence of gastroduodenal ulcers, especially in those with a history of gastric ulcers. Serious comorbidities, particularly cardiovascular, raise in-hospital mortality risk from NSAID ulcer complications, mitigated by proton pump inhibitors. Approximately 50% of Japanese gastroduodenal ulcers with complications are NSAID-related, emphasizing the need for preventive measures based on risk factors [12,20,21,22].

### 3.4. Other Causes of Gastroduodenal Perforation

There are other factors recognized to be the cause of GDP, as shown in Table 4. Among the 1558 patients who had a renal transplant, the prevalence of developing GDP ranged from 4.7% to 37% [23,24,25,26]. Furthermore, 7.1% and 2.4% of patients undergoing major abdominal surgery and neurosurgery had GDP as postoperative complications [27,28]. In patients with cardiovascular disease, GDP was one complication that occasionally occurred, with 1.2 percent of 17,598 patients having upper gastrointestinal complications such as bleeding, ulcer, or perforation for both the placebo and pantoprazole treatment groups [29,30,31]. Complications also occurred after cardiovascular surgery with cardiopulmonary bypass, and gastroduodenal ulcer perforation occurred in 27.3% of 2349 patients [29]. Among 39 patients who had laparoscopic surgery due to hernia; 35% of patients experienced GDP [32], and it also developed in 23% of patients with colorectal cancer as the side effect of chemotherapy [33]. In intestinal transplantation cases, six children out of fifty-nine had spontaneous bowel rupture (13%) [34]. Among other causes, such as burn, opiate addiction, and smoking, gastroduodenal perforation occurred as a side effect [35,36,37]. However, prophylaxis was deemed important to prevent a more severe gastroduodenal event [11,38].

### 3.5. Gastroduodenal Perforation Treatment (Laparoscopy vs. Open Surgery)

The frequency of laparoscopy’s success rate is shown in Table 5. When compared to open surgery, laparoscopic procedures have a much higher success rate, with approximately a mean of 94.4% from a total of 555 patients. Success rates were obtained from the difference between total patients and mortality. Most of the time, the laparoscopic treatment showed no or little complications, wound infections, sepsis, stenosis, and extra-abdominal issues [13,15,40,41,42,43,44,45,46]. Despite the great result of laparoscopic outcomes, some failures, morbidity, and mortality occur in small numbers of patients due to delayed treatment, old age, comorbidity, and other reasons that are unrelated to gastroduodenal perforation [14,16]. However, in a few instances where there was immediate leakage and bleeding, reoperations were also performed [47]. The laparoscopic treatment of patients with ulcers resulted in good cicatrisation, and no pyloric stenosis remained [40].

Even though laparoscopic surgery has shown a greater success rate, open surgical procedures are still widely used to treat perforated gastroduodenal ulcers. As many as 30,919 patients in this study were treated with open surgery, with an average success rate of 85.03% (Table 5). Even though open surgery was more frequently performed, open surgery has a higher risk of mortality compared to laparoscopic surgery. The Ohene-Yeboah study showed that the incidence of mortality was 9.9% after surgery as a result of severe intra-abdominal sepsis and multi-organ failure [49]. Another study also showed that the mortality rate in perforated gastric cancer after surgery was 13.8% [50]. Other contributing factors, such as the buildup of fat, advancing age, the presence of other medical conditions, lack of appropriate therapy, and impaired liver function, have been found to elevate the risk of postoperative complications and mortality [48,52,56,58,59].

## 4. Discussion

Previous studies showed that *H. pylori*’s urease, flagella, chemotaxis system, and adhesins are responsible for establishing colonization and exacerbated clinical outcomes [60]. Moreover, studies showed that several virulence factors such as outer inflammatory protein (OipA), epithelial gene A1 (iceA1), babA2-gene positive (encodes BabA protein), duodenal ulcer promoting gene cluster (dupA cluster), vacA s1/m1 genotype, and cagA-gene positive expression are associated with peptic ulcer disease [61]. Our study showed that approximately 58% of patients with gastroduodenal perforation were infected with *H. pylori*, which is consistent with previously reported studies [62]. Patients afflicted with *H. pylori* should undergo treatment to eradicate infection and avoid its reoccurrence, hence averting the development of gastrointestinal diseases [2]. The simultaneous administration of many antibiotics, including amoxicillin, clarithromycin, and ceftriaxone, is typically employed to eliminate *H. pylori* bacteria. This treatment is sometimes combined with a proton pump inhibitor (PPI) and omeprazole [7,13,17]. The length of antibiotic treatment and abdominal drainage was contingent upon the clinical results. The duration of antibiotic medication may be extended due to a persistent fever, stomach discomfort, and intestinal paralysis [13]. Perforated, *H. pylori*-negative chronic duodenal ulcers or recurrent ulcers may be treated with final anti-ulcer surgery (parietal cell vagotomy anterior linear gastrectomy). Surgeries are often the first line of treatment for perforations, especially for patients who are in a critical condition and need immediate medical attention [16].

*Helicobacter pylori* infection is a major contributor to developing GDP, although other variables, such as the utilization of NSAIDs, also play a part in this process. Non-steroidal anti-inflammatory drugs are characterized by their selectivity in inhibiting COX enzymes. Ibuprofen, naproxen, and diclofenac, which suppress both COX-1 and COX-2, can interfere with COX-1’s protective mechanisms in the stomach lining, increasing the risk of perforation and ulcers [63]. Selective COX-2 inhibitors like celecoxib and rofecoxib were developed to minimize gastrointestinal problems from non-selective NSAIDs. They reduce stomach ulcers and perforation risk compared to non-selective NSAIDs [19]. However, certain COX-2 inhibitors may increase cardiovascular risks. The company took rofecoxib off the market on its own in September 2004 because of worries about the higher chance of heart attacks and strokes that came with its long-term, high-dose use. This is said to have caused between 88,000 and 140,000 cases of serious heart disease [64].

Our study showed that the overall prevalence of GDP among NSAID users was considerably low, while the prevalence of *H. pylori* among patients with GDP was relatively high. This finding is consistent with earlier studies that reported, despite the risk of gastroduodenal damage among NSAID users, the chance of perforation being low when taking a low dose of NSAIDs [65]. Gastroduodenal perforation can also occur as a side effect of other medical therapies and/or surgeries. Nevertheless, the prevalence of GDP among such patients remains low, except for those who engage in smoking and suffer from opiate addiction since they are more vulnerable to GDP [35,36]. Although NSAIDs and other therapies may play a role in the development of peptic ulcer disease, gastric cancer, malt lymphoma, and gastroduodenal perforation, the infection of *H. pylori* should not be overlooked.

Patients with gastric perforation need prompt treatment to prevent mortality. Surgical interventions are often undertaken as an emergency to seal leakages, followed by an additional regimen for treating the *H. pylori* infection. Open surgery and laparoscopic procedures are the most frequently performed for gastric perforation treatment. However, open surgery and laparoscopy trends are shifting. The 1998–2012 investigation revealed that the number of patients that underwent open surgery decreased after 2004, while the number of patients who underwent laparoscopy increased [46]. Although the use of laparoscopic procedures is growing, open surgery is still often employed to treat patients with perforated gastroduodenal conditions for several reasons [15,17]. Open surgeries may still be necessary to treat patients with gastric perforation based on circumstances such as the severity of the perforation, hemodynamic instability, or the presence of significant contamination. Open surgery enables a comprehensive examination of the abdominal cavity, which helps in effectively preventing contamination and managing difficult perforations optimally [66].

Taken together, this study acknowledges that the collective impact of various causes may contribute to stress on the gastrointestinal system, leading to ulcer perforation. However, *H. pylori* infection also plays a significant role in gastroduodenal perforation with or without NSAID use or due to the consequence of surgeries or the use of chemotherapy and transplantation. To prevent gastric perforation, it is necessary to identify and manage risk factors while implementing evidence-based therapy. Peptic ulcers, which can lead to stomach ruptures, can be averted by specifically addressing the *H. pylori* infection using medicines. Non-steroidal anti-inflammatory drug utilization necessitates meticulous management, encompassing the evaluation of potential risks and the prescription of prophylactic medications or other methods of pain management. Restricting the consumption of alcohol and tobacco; adhering to a diet abundant in nutrients from fruits, vegetables, and whole grains; and controlling portion sizes might enhance the wellbeing of the stomach. Meditation and yoga alleviate chronic stress, a potential trigger for stomach ulcers. Proton pump inhibitors (PPIs) and histamine-2 blockers aid in the healing and prevention of ulcers by maintaining the integrity of the mucosal lining. Regular medical examinations aid in the identification and treatment of disorders that can lead to stomach perforation. Preventive strategies for maintaining a healthy weight include engaging in regular exercise, following a balanced diet, and refraining from the use of gastric-irritating medications. Timely identification and management of gastrointestinal symptoms is crucial in order to prevent stomach perforation. Healthcare experts can avoid gastric perforation by applying these scientific principles in a customized manner. However, if gastric perforation occurs, certain procedures, such as laparoscopic or open surgery, are necessary to be performed immediately.

## 5. Study Limitation

It is imperative to recognize specific constraints in our research that could potentially impact the interpretation of our findings. An initial consideration must be given to the possibility that potential biases, such as recall bias or selection bias, may compromise the validity of our findings. Furthermore, discrepancies in study designs across various research components could potentially introduce variability in the collected data. The potential impact of data acquisition limitations, including insufficient sample size or missing data points, on the applicability of our findings should not be overlooked. Despite our best efforts to mitigate these constraints, it is important to acknowledge their existence when interpreting the results of this study. It is advisable that future investigations incorporate more substantial and varied sample sizes, as well as rigorous study designs, in order to augment the validity and applicability of our results.

## Figures and Tables

**Table 1:** Study selection criteria.

Criteria
Inclusion Criteria
Retrospective and prospective studies. The necessary data were provided. Evidence comprises gastroduodenal perforation, bleeding, and ulcers. Treatment of gastroduodenal perforation. Patients were followed up, and results were reported.
Exclusion Criteria
The literature was not the original article (such as meta-analysis, review), or a literature duplication. The object of the study was cellular-based or animal-based. The evidence occurred other than in the gastric or duodenal area. The articles were not in English.

**Table 2:** The prevalence of *H. pylori* infection among patients with gastroduodenal perforation.

No.	Study	Topic	Total Number of Patients	*H. pylori* Prevalence (*n*)
1	Tokunaga et al. (1998) [8]	Impact of *H. pylori* in the severity of a perforated ulcer.	113	92% (104)
2	Debongnie et al. (1995) [9]	Recurrence of gastric perforation in patients infected with *H. pylori*.	36	56% (20)
3	Casali et al. (2012) [10]	*H. pylori* test in patient with gastric perforation.	14	84.6% (12)
4	Thirupathaiah et al. (2020) [3]	The association between *H. pylori* and a perforated gastroduodenal ulcer.	48	12.5% (6)
5	Ha et al. (2016) [11]	The gastrointestinal safety of herbal medicine in patients with osteoarthritis.	761	53% (403)
6	Vonkeman et al. (2007) [12]	PPI reduced the risk of NSAID ulcer complications.	104	20% (21)
7	Köninger et al. (2004) [13]	Laparoscopic repair of a perforated gastroduodenal ulcer.	20	65% (13)
8	Agaba et al. (2016) [14]	The incidence of complicated PUD and analyses changes in surgical management.	400	84% (336)
9	Pescatore et al. (1998) [3]	A new method combining laparoscopy and endoluminal endoscopy was designed to ensure complete closure of the perforation.	6	100% (2)
10	Okidi et al. (2020) [17]	An observational study of patients with suspected NTGDP who had exploratory laparotomy during a one-year period.	29	13.8% (4)
11	Yan et al. (2019) [15]	Gastroduodenal perforation in children: causes, symptoms, and customized surgical care.	20	30% (6)
12	Kujath et al. (2002) [16]	Perforated gastroduodenal ulcers: surgical management and prognosis.	102	26.3% (27)

**Table 3:** Gastroduodenal events induced by non-steroidal anti-inflammatory drugs.

No.	Study	Drugs	Study Design	Country	Total Studied Patients	Gastroduodenal Perforation (%, Number)	RR (95% CI)
1	Bombardier et al. (2000) [19]	Rofecoxib and naproxen	Cohort	Twenty-two countries.	8076	6.6% (533)	0.4 (0.2–0.8)
2	Yeomans et al. (2017) [20]	Colecoxib, ibuprofen, and naproxen	Cohort	United States, Canada, Australia, Brazil, Colombia, Costa Rica, Mexico, Panama, Peru, Philippines, Taiwan, Hong Kong, and Ukraine.	24,081	1.7% (409)	0.44 (0.28–0.7)
3	Cheatum et al. (1999) [22]	>5 NSAID	Cross sectional	Fourteen countries that abided by the Declaration of Helsinki.	1826	37.1% (676)	0.811 (0.067–3.4)
4	Ishikawa et al. (2012) [21]	>5 NSAID	Consecutive cross sectional	Japan	305	50% (153)	1.715 (1.98–18.89)
5	Vonkeman et al. (2007) [12]	>5 NSAID	Cohort	Germany	10,402	1% (104)	0.498 (0.23–0.78)

**Table 4:** Other risk factors associated with a gastroduodenal event.

No.	Study	Risk	Treatments	Total Number of Patients	Prevalence of Gastroduodenal Perforation (*n*)
1	Ahonen et al. (1977) [25]	Renal transplantation	Antacids	434	10.3% (45)
2	Stuart et al. (1981) [30]	Renal transplantation	Antacids	167	4.7% (8)
3	Benoit et al. (1993) [23]	Renal transplantation	Immuno-suppressant, steroids	614	16.2% (99)
4	Meyers et al. (1979) [24]	Renal transplantation	Steroids	343	37% (127)
5	Gupte et al. (2010) [34]	Intestinal transplantation	Immunosuppressive regimen and cyto-megalo-virus (CMV) prophylaxis	46	13% (6)
6	Boufi et al. (2013) [28]	Pancreatic duodenectomy	Octreotide	14	7% (1)
7	Chan and Mann (1989) [27]	Neurosurgical	Dexamethasone and cimetidine	381	2.4% (9)
8	Bhatt et al. (2010) [30]	Coronary artery disease	Clopidogrel in combination with either omeprazole or placebo	3761	1.1% (42)
9	Moayyedi et al. (2019) [31]	Cardiovascular and peripheral artery disease	Rivaroxaban, aspirin, and pantoprazole	8791	1.2% (106)
10	Dong et al. (2012) [29]	Cardiovascular operations	Cardiopulmonary bypass	2349	6.1% (144)
11	Geisel et al. (2014) [39]	Biliodigestive anastomosis	Bilobar or unilobar treatment	143	6.29% (9)
12	Pomar et al. (2010) [32]	Hernia surgery	NA	39	20.5% (8)
13	Ogata et al. (1996) [33]	Colorectal cancer	Chemotherapy	43	23% (10)
14	Simons et al. (1995) [38]	Stress ulceration	NA	33637	0.05% (17)
15	Mc Alhany et al. (1976) [37]	Burn	Antacid	24	29.1% (7)
16	Kahrom and Kahrom (2010) [36]	Opiate addiction	NA	35	100% (35)
17	Svanes et al. (1997) [35]	Smoking	NA	175	96% (168)

**Table 5:** Laparoscopic and open surgery treatment for gastroduodenal perforation incidence.

No.	Study	Total Number of Patients	Mortality Rate (*n*)	Success Rate	Outcome
Laparoscopy					
1	Kujath et al. (2002) [16]	102	22.5% (23)	78.33%	Failure and mortality in postoperative patients are mostly attributable to delayed treatment, old age, and comorbidities.
2	Yan et al. (2019) [15]	7	0	100%	There were no complication issues among the individuals.
3	Koninger et al. (2004) [13]	20	0	100%	There was no insufficiency, wound infection, stenosis, or persistent peritonitis.
4	Costalat and Alquier (1995) [40]	15	0	100%	The ulcer had excellent cicatrisation and no pyloric stenosis persisted.
5	Cocorullo et al. (2016) [41]	75	0	100%	No morbidity and mortality found.
6	Žáček et al. (2014) [42]	110	1	99.1%	Total morbidity was 10.9% after laparoscopic surgery, and no wound infection was detected.
7	Laforgia et al. (2017) [47]	21	0	100%	Reoperations were necessary due to three instances of leakage and one instance of bleeding.
8	Reusen et al. (2017) [43]	5	0	100%	There were no deaths, conversions, extra-abdominal issues, or wound infections.
9	Agaba et al. (2016) [14]	48	2% (1)	98%	One patient who had laparoscopic repair passed away from reasons unrelated to gastroduodenal perforation.
10	Harvitkar et al. (2021) [44]	9	0	100%	In the first two weeks after surgery, 16% of patients had minor complications, including trocar wound infections, terminal ileum typhoid perforations, and moderate paralytic ileus. None of these patients reported suture or staple leakage after surgery.
11	Navez et al. (1998) [45]	69	4% (3)	(66) 96.1%	There was no occurrence of malignant hypercapnia, and 0.9% of patients survived postoperative septic shock.
12	Kirshtein et al. (2005) [46]	68	5% (3)	(65) 96%	Sepsis, sub-hepatic abscess, and pulmonary problems occur in only a small number of patients.
13	Pescatore et al. (1998) [7]	6	0	100%	There was no morbidity and no mortality
	Total number of patients	555	5.5% (31)	94.4% (524)	
Open Surgery					
1	Okidi et al. (2020) [17]	29	34.5% (10)	65.5%	Preoperative pyrexia, delay, shock, and peritoneal contamination were all associated with higher fatality rates.
2	Yan et al. (2019) [15]	13	0	100%	There were no patients with complication issues.
3	Hasselager et al. (2016) [48]	4086	30.8% (1258)	69.25%	Patients who were overweight, have a history of multiple diseases, and have a severe condition are at a greater risk of needing an additional operation.
4	Ohene-Yeboah (2006) [49]	3114	23.7% (738)	76.3%	Acute appendicitis, typhoid ileal perforation, acute intestinal obstruction, and gastroduodenal perforation were the most frequent abdominal admissions.
5	Wang et al. (2017) [50]	2738	13.8% (378)	86.2%	Emergency peritonitis therapy and vigorous gastric cancer surgery may improve the acute and oncologic outcomes of patients with perforated gastric cancer.
6	Chao et al. (1999) [51]	11	18.2% (2)	81.8%	Perforation of the gastroduodenal junction in patients with cancer who were not receiving therapy led to acceptable short-term surgical results.
7	Tsugawa et al. (2001) [52]	130	26.7% (35)	73.3%	Older people have poorer prognoses. Due to low mortality and minimal stress, a simple closure and vagotomy is appropriate for duodenal ulcers, particularly in persons with a 20 mm perforation or severe duodenal stenosis. Because of its low recurrence rate, gastroplasties are sometimes advised for stomach ulcers.
8	Maeda et al. (2022) [53]	16,209	8.8% (1426)	91.2%	In Japan, the level of care offered by emergency surgical operations is stable in terms of mortality rate throughout the week.
9	Jordan and Debakey (1963) [54]	496	5.4% (27)	94.6%	In appropriately chosen patients, the final surgical treatment should be used as the operation of choice.
10	Lehnert and Herfarth (1993) [55]	69	29% (20)	71%	To improve treatment outcomes, blood products (particularly coagulation factors) should be replenished early and in suitable volumes, and operating operations should be limited to ulcer control.
11	Anwar et al. (1996) [56]	32	25% (8)	75%	Schistosomal portal hypertension makes peptic ulcer disease hazardous. Emergency treatments, postoperative issues, and patients with modified Child B constitute to increased mortality; liver function must be regulated preoperatively to avoid surgical complications and hepatic decompensation.
12	Agaba et al. (2016) [14]	352	2% (8)	98%	If morbidity and death rates are to be reduced, an early surgical intervention is advised. In the majority of cases, simple closure with *H. pylori* eradication and acid suppression will be sufficient.
13	Agarwal et al. (2017) [57]	20	20% (4)	80%	Triple tube drainage for problematic gastroduodenal perforations is practical, simple in emergencies, and enables patients to recover in 2–3 weeks. It eliminates technically difficult and risky operations.
14	Schroder et al. (2014) [58]	3611	19.6% (708)	80.4%	Patients with perforated peptic ulcers need basic treatment. In patients with intractable ulcer bleeding, vagotomy/drainage had a lower postoperative death rate than ulcer oversew.
15	Chao et al. (1998) [59]	9	44.4% (4)	55.6%	Early treatment of cancer patients with spontaneous gastroduodenal perforation with a high index of suspicion of the illness may enhance survival.

## Data Availability

Not applicable.
